# Effect of exogenous estrogens and progestogens on the course of migraine during reproductive age: a consensus statement by the European Headache Federation (EHF) and the European Society of Contraception and Reproductive Health (ESCRH)

**DOI:** 10.1186/s10194-018-0896-5

**Published:** 2018-08-31

**Authors:** Simona Sacco, Gabriele S. Merki-Feld, Karen Lehrmann Ægidius, Johannes Bitzer, Marianne Canonico, Andreas R. Gantenbein, Tobias Kurth, Christian Lampl, Øjvind Lidegaard, E. Anne MacGregor, Antoinette MaassenVanDenBrink, Dimos-Dimitrios Mitsikostas, Rossella Elena Nappi, George Ntaios, Koen Paemeleire, Per Morten Sandset, Gisela Marie Terwindt, Kjersti Grøtta Vetvik, Paolo Martelletti, on behalf of the European Headache Federation (EHF), the European Society of Contraception and Reproductive Health (ESCRH)

**Affiliations:** 1 Department of Applied Clinical Sciences and Biotechnology, University of L’Aquila, L’Aquila, Italy; 2 Clinic for Reproductive Endocrinology, Department of Gynecology, University Hospital, Zürich, Switzerland; 3 Department of Neurology, Bispebjerg Hospital and University of Copenhagen, Copenhagen, Denmark; 4 Department of Obstetrics and Gynecology, University Hospital of Basel, Basel, Switzerland; 5 Université Paris-Saclay, University Paris-Sud, UVSQ, CESP, Inserm UMRS1018, Paris, France; 6 Neurology & Neurorehabilitation, RehaClinic, Bad Zurzach, University of Zurich, Zürich, Switzerland; 7 Institute of Public Health, Charité - Universitätsmedizin Berlin, Berlin, Germany; 8 Headache Medical Center Seilerstaette Linz, Linz, Austria; 9 Department of Geriatric Medicine Ordensklinikum Linz, Linz, Austria; 10 Department of Obstetrics & Gynaecology, Rigshospitalet, Faculty of Health Sciences, University of Copenhagen, Copenhagen, Denmark; 11 Centre for Neuroscience & Trauma, BICMS, Barts and the London School of Medicine and Dentistry, London, UK; 12 Barts Health NHS Trust, London, UK; 13 Erasmus Medical Center Rotterdam, Department of Internal Medicine, Division of Vascular Medicine and Pharmacology, Rotterdam, The Netherlands; 14 Department of Neurology, University of Athens, Athens, Greece; 15 Research Centre for Reproductive Medicine, Gynecological Endocrinology and Menopause, IRCCS S. Matteo Foundation, Department of Clinical, Surgical, Diagnostic and Pediatric Sciences, University of Pavia, Pavia, Italy; 16 University Consortium for Adaptive Disorders and Head Pain (UCADH), University of Pavia, Pavia, Italy; 17 Department of Medicine, University of Thessaly, Larissa, Greece; 18 Department of Neurology, Ghent University Hospital, Ghent, Belgium; 19 University Hospital Rikshospitalet, University of Oslo, Oslo, Norway; 20 Department of Neurology, Leiden University Medical Center, Leiden, the Netherlands; 21 Department of Neurology, Akershus University Hospital, Lørenskog, Norway; 22 Department of Clinical and Molecular Medicine, Sapienza University, Rome, Italy

**Keywords:** Migraine, Headache, Estrogens, Progestogens, Hormonal contraceptives, Contraception

## Abstract

We systematically reviewed data about the effect of exogenous estrogens and progestogens on the course of migraine during reproductive age. Thereafter a consensus procedure among international experts was undertaken to develop statements to support clinical decision making, in terms of possible effects on migraine course of exogenous estrogens and progestogens and on possible treatment of headache associated with the use or with the withdrawal of hormones. Overall, quality of current evidence is low. Recommendations are provided for all the compounds with available evidence including the conventional 21/7 combined hormonal contraception, the desogestrel only oral pill, combined oral contraceptives with shortened pill-free interval, combined oral contraceptives with estradiol supplementation during the pill-free interval, extended regimen of combined hormonal contraceptive with pill or patch, combined hormonal contraceptive vaginal ring, transdermal estradiol supplementation with gel, transdermal estradiol supplementation with patch, subcutaneous estrogen implant with cyclical oral progestogen. As the quality of available data is poor, further research is needed on this topic to improve the knowledge about the use of estrogens and progestogens in women with migraine. There is a need for better management of headaches related to the use of hormones or their withdrawal.

## Introduction

The role of female hormones in the pathogenesis of migraine is well-recognized [1, 2]. Migraine is more prevalent in women than in men, it usually starts after puberty and in many women improves during pregnancy and after the menopause [1, 3, 4]. The menstrual phase of the female cycle represents a trigger for migraine attacks in many women [1, 4]. Additionally, exogenous hormones may change the course of migraine by inducing de novo migraine, inducing de novo aura, worsening previous migraine but also improving migraine particularly those attacks related to menstruation [5, 6].

The criteria to diagnose migraine related to menstruation (Table 1) and to diagnose headache related to the use and to the withdrawal of hormones (Table 2) have varied over years [7–10]. The first version of the International Classification of Headache Disorders (ICHD), 1988, did not report any formal criteria for migraine related to menstruation [7]. The authors recognized that in some women, migraine without aura (MO) may be almost exclusively linked with menstruation, so called menstrual migraine (MM), and indicated that it seemed reasonable to require that 90% attacks should occur between two days before menses and the last day of menses. Appendix criteria for migraine related to menstruation were introduced in 2004, with the second edition of the ICHD (Table 1) [8]. Two entities were recognised: pure menstrual migraine (PMM) where attacks are exclusively related to menstruation; menstrually related migraine (MRM) where attacks occur additionally at other times of the cycle. Both were forms of MO, along with non-menstrual MO or migraine with aura (MA) in the case of MRM. PMM with aura and MRM with aura are new entries in the Appendix of the classification system available since 2018 [10].

**Table 1:** Diagnostic criteria of migraine related to menstruation according to the different editions of the International Classification of Headache Disorders (ICHD)

ICHD, II edition, 2004	
A.1.1.1 Pure menstrual migraine without aura	
A. Attacks, in a menstruating woman, fulfilling criteria for 1.1 Migraine without aura B. Attacks occur exclusively on day 1 ± 2 (i.e. days − 2 to + 3) of menstruation in at least two out of three menstrual cycles and at no other times of the cycle	
A.1.1.2 Menstrually-related migraine without aura	
A. Attacks, in a menstruating woman, fulfilling criteria for 1.1 Migraine without aura B. Attacks occur on day 1 ± 2 (i.e. days −2 to + 3) of menstruation in at least two out of three menstrual cycles and additionally at other times of the cycle	
ICHD, III edition, beta version, 2013	
A1.1.1 Pure menstrual migraine without aura	
A. Attacks, in a menstruating woman, fulfilling criteria for 1.1 Migraine without aura and criterion B below B. Documented and prospectively recorded evidence over at least three consecutive cycles has confirmed that attacks occur exclusively on day 1 ± 2 (i.e. days −2 to + 3) of menstruation in at least two out of three menstrual cycles and at no other times of the cycle	
A1.1.2 Menstrually related migraine without aura	
A. Attacks, in a menstruating woman, fulfilling criteria for 1.1 Migraine without aura and criterion B below B. Documented and prospectively recorded evidence over at least three consecutive cycles has confirmed that attacks occur on day 1 ± 2 (i.e. days −2 to + 3) of menstruation in at least two out of three menstrual cycles, and additionally at other times of the cycle	
ICHD, III edition, 2018	
A1.1.1 Pure menstrual migraine without aura	
A. Attacks, in a menstruating woman, fulfilling criteria for 1.1 Migraine without aura and criterion B below B. Occurring exclusively on day 1 ± 2 (i.e. days −2 to + 3) of menstruation in at least two out of three menstrual cycles and at no other times of the cycle	
A1.1.2 Menstrually related migraine without aura	
A. Attacks, in a menstruating woman, fulfilling criteria for 1.1 Migraine without aura and criterion B below B. Occurring on day 1 ± 2 (i.e. days −2 to + 3) of menstruation in at least two out of three menstrual cycles, and additionally at other times of the cycle	
A1.2.0.1 Pure menstrual migraine with aura	
A. Attacks, in a menstruating woman, fulfilling criteria for 1.2 Migraine with aura and criterion B below B. Occurring exclusively on day 1 ± 2 (i.e. days −2 to + 3) of menstruation in at least two out of three menstrual cycles and at no other times of the cycle	
A1.2.0.2 Menstrually related migraine with aura	
A. Attacks, in a menstruating woman, fulfilling criteria for 1.2 Migraine with aura and criterion B below B. Occurring on day 1 ± 2 (i.e. days −2 to + 3) of menstruation in at least two out of three menstrual cycles, and additionally at other times of the cycle

**Table 2:** Diagnostic criteria of headache related to the use and to the withdrawal of hormones according to the different editions of the International Classification of Headache Disorders (ICHD)

ICHD, II edition, 2004	
8.3.1 Exogenous hormone-induced headache	
A. Headache or migraine fulfilling criteria C and D B. Regular use of exogenous hormones C. Headache or migraine develops or markedly worsens within 3 months of commencing exogenous hormones D. Headache or migraine resolves or reverts to its previous pattern within 3 months after total discontinuation of exogenous hormones	
8.4.3 Estrogen-withdrawal headache	
A. Headache or migraine fulfilling criteria C and D B. Daily use of exogenous estrogen for ≥3 weeks, which is interrupted C. Headache or migraine develops within 5 days after last use of estrogen D. Headache or migraine resolves within 3 days	
ICHD, III edition, beta version, 2013	
8.1.12 Headache attributed to exogenous hormone	
A. Any headache fulfilling criterion C B. Regular intake of one or more exogenous hormones C. Evidence of causation demonstrated by both of the following: 1. Headache has developed in temporal relationship with the commencement of hormone intake 2. One or more of the following: a) headache has significantly worsened after an increase in the dosage of the hormone b) headache has significantly improved or resolved after a reduction in the dosage of the hormone c) headache has resolved after cessation of hormone intake d) Not better accounted for by another ICHD-3 diagnosis	
8.3.3 Estrogen-withdrawal headache	
A. Headache or migraine fulfilling criterion C B. Daily use of exogenous estrogen for ≥3 weeks, which has been interrupted C. Evidence of causation demonstrated by both of the following: 1. headache or migraine has developed within five days after the last use of estrogen 2. headache or migraine has resolved within three days of its onset D. Not better accounted for by another ICHD-3 diagnosis	
ICHD, III edition, 2018	
8.1.10 Headache attributed to long-term use of non-headache medication	
A. Headache present on ≥15 days/month and fulfilling criterion C B. Long-term use of a medication has occurred for purposes other than the treatment of headache C. Evidence of causation demonstrated by at least two of the following: 1. headache has developed in temporal relation to the commencement of medication intake 2. one or more of the following: a) headache has significantly worsened after an increase in dosage of the medication b) headache has significantly improved or resolved after a reduction in dosage of the medication c) headache has resolved after cessation of the medication 3. the medication is recognized to cause head- ache, in at least some people, during long- term use D. Not better accounted for by another ICHD-3 diagnosis	
8.3.3 Estrogen-withdrawal headache	
A. Headache or migraine fulfilling criterion C B. Daily use of exogenous estrogen for ≥3 weeks, which has been interrupted C. Evidence of causation demonstrated by both of the following: 1. headache or migraine has developed within five days after the last use of estrogen 2. headache or migraine has resolved within three days of its onset D. Not better accounted for by another ICHD-3 diagnosis	

Since the first ICHD classification [7], it was recognized that headache may be attributable to the use of substances or their withdrawal but formal categories referring to estrogens were introduced in the second edition of the ICHD (Table 2) [8]. Headache attributable to hormones, both in the second and the third edition (beta) of the ICHD, could be diagnosed in the presence of either new onset headache or of worsening of pre-existing headache [8, 9]. Criteria also required resolution or return to the previous pattern after cessation of the hormones. A significant change was introduced in 2018 where the diagnosis of headache attributable to exogenous hormones requires the presence of headache for at least 15 days per month [10]. At variance, the diagnosis of estrogen-withdrawal headache remained substantially unchanged over years and requires the onset of headache, in women who have been taking estrogens for three weeks or longer, within 5 days from estrogen withdrawal. However, evidence for the duration of treatment with estrogen before withdrawal headache occurs is lacking.

Several attempts were made to manipulate the female hormonal cycle to try to improve migraine [6, 11]. Studies have investigated both MO, MA and migraine attacks related to menstruation [6, 11]. Additionally, as in users of combined hormonal contraceptives (CHC) migraine attacks mostly occur during the hormone free interval, studies also evaluated how different estrogen or progestogen regimens impact on the course of migraine [12–15]. CHC have been associated with an increased risk of ischemic stroke in women with migraine [16–21]. A working group including headache experts, gynaecologists, stroke experts, and epidemiologists developed a first consensus document about the safety of hormonal contraceptives (HCs) in female migraineurs of reproductive age [22]. According to the recommendations of the European Headache Federation (EHF)/European Society of Contraception and Reproductive Health (ESCRH) consensus group, CHCs should not be used in all women with MA and women with MO who have additional risk factors. Progestogen-only hormonal contraceptives (PHCs) can be safely considered in this group of patients [22]. Currently, no formal guidelines specifically address hormonal treatment of migraine. The aim of this consensus document is to provide recommendations on the management of migraine with the use of estrogens and progestogens in women of reproductive age.

## Methods

In July 2017, EHF representatives selected a panel of international multidisciplinary experts in migraine and hormonal contraception (HC). The panel was chosen to represent the breadth of knowledge and experience and a wide variety of opinions internationally. The aim of this statement is to provide evidence-based guidance to clinicians about evidence-based options for the management of migraine with exogenous estrogens and progestogens.

### Review of the literature

A systematic review of the literature was performed according to the Preferred Reporting Items for Systematic Reviews and Meta-Analyses (PRISMA) guidelines [23]. We identified key papers on possible benefits of the use of estrogens and progestogens in migraine. An initial literature search included all papers indexed on PubMed and Scopus, from inception to October 23, 2017. The systematic literature search was repeated at the end of the consensus procedure to include all relevant papers published until May 2018. The following search string was used in both databases: “migraine AND (hormone OR estrog* OR progest* OR contracept*)”. Two investigators independently screened the titles and abstracts of the publications identified to verify study eligibility. Literature screening was conducted in two steps. In the first step, studies were excluded after reading the title and the abstract for clear exclusion criteria. For studies that passed the first step, the full text was assessed to decide inclusion/exclusion. Disagreements were resolved by consensus. The reference lists and Google Scholar citations of the selected articles were also screened. The reasons for exclusion were recorded and summarized. To summarize the search results, a data extraction sheet was developed including the information of interest. Papers retrieved from the literature search as well as summary tables were shared among the panelists.

### Data extraction

A general description of the study was extracted for each publication. We extracted first author name and year of publication, full citation, study design and setting, study period, number of included patients, diagnostic criteria for migraine, migraine type, treatments type, duration of observations and treatments, study results. Data extraction was performed by a single researcher (SS) and double checked.

### Inclusion and exclusion criteria

Inclusion and exclusion criteria were selected prior to the literature search.

We included studies that were 1) observational (retrospective and prospective) or interventional and in which an estrogen and/or a progestogen drug was assessed as possible treatment strategy for migraine; 2) were published in English or in other languages if a reliable translation could be obtained; 3) using reliable criteria to diagnose migraine; 4) assessing treatment with any form of estrogen or progestogen; 5) reporting any outcome referring to migraine frequency, severity, duration, disability, or use of drugs to treat the acute attacks before and after treatment or in treated and untreated women. Whenever different studies referring to the same population of patients were available we included the study with the largest population or the longest follow-up. We excluded studies 1) with observational designs not reporting outcome before and after treatment or not comparing at least two treatment strategies; 2) using estrogen or progestogen that is no longer available or that is not considered a feasible strategy; 3) performed in post-menopausal women.

### Quality assessment

For each of the selected studies one author (SS) addressed the quality of evidence. The quality of evidence was addressed according to GRADE approach for single studies [24]. Randomized trials were considered as high quality of evidence but their quality was downgraded in the case of study limitations such as lack of allocation concealment, lack of blinding, incomplete accounting for patients and outcome events, selective outcome reporting bias, or other limitations such as inadequate sample or lack of sample size calculation [25]. Observational studies were considered as low quality of evidence but their quality was upgraded if large magnitude effects, dose-response gradient, if plausible confounding can increase confidence in the estimate or other considerations [26].

### Development of the expert consensus

The consensus process was performed according to the Delphi method [27]. Development of the consensus statement was organized in three rounds. In each round, panelists were instructed not to discuss among themselves and to send their feedback only to the facilitator (SS). Two core panelists (SS, PM) developed a draft document containing the statements. In round 1, the draft containing the statements was sent by e-mail to all panelists accompanied by a clear explanation of the objectives of the study and specific instructions. Panelists were asked to provide free-text comments to all the statements and to suggest additional items of relevance or questions to be answered. Thereafter, the facilitator analysed answers obtained during round 1 and drafted a revision version of the statements with additional items. In round 2, a further draft of the documents and of the statements was sent by e-mail to all panelists. Each panelist was asked to report their agreement for each statement and provide suggestions. Panelists were also given the opportunity to identify further additional items not included in the initial list of statements. Responses were then analysed by the facilitator and used to refine statements. In round 3, a revised draft of the document and of the statements was developed and emailed to all panelists and the panelists were asked again to revise and to express their agreement. The panelists were also required to provide a rank order of the statements. Response frequencies for each item were calculated and entered anonymously into a database. Statements to be included in the final document required at least 80% agreement from the panel.

### Drafting of the statements

Quality of evidence and strength of the recommendations were rated according to the American College of Chest Physicians Task Force [28]. We also used the suggestions provided by the ACCP referring to wording of the recommendations. When making a strong recommendation we used the terminology “We recommend…”, whereas when making a weak recommendation, less definitive wording was used, such as, “We suggest…”.

## Results

For the present consensus statement, we adopt the diagnoses of PMM or MRM as defined in the selected studies. The term MM is used to encompass both PMM and MRM.

**Fig. 1:**
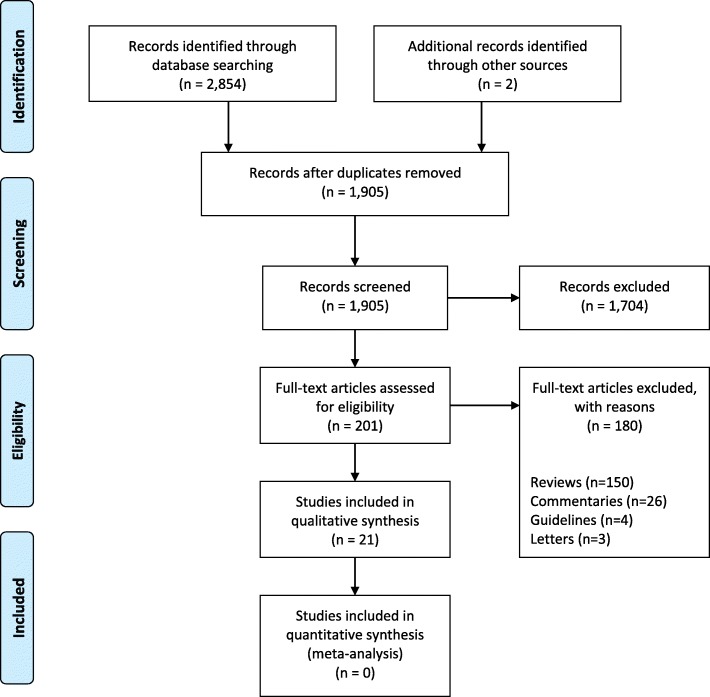
Flow-chart for the systematic review. Search string: “migraine AND (hormone OR estrog* OR progest* OR contracept*)”. Date: 23-Oct-2017

We found 21 studies which evaluated the effects of estrogens and progestogens on headache in women of reproductive age (Fig. 1) [12–15, 29–45].

In 11 studies treatment was specifically used for headache prevention [12, 29–38], in 8 studies treatment effect on headache was evaluated in women who required treatment for contraception or medical reasons [13–15, 39–43], in 2 studies it was not specifically stated if treatment was prescribed specifically for headache treatment or for other indications [44, 45]. Five studies were performed in women with MO or MA not necessarily related to menstruation [12–14, 41, 43], 10 in MRM or MM [29–39, 43, 45], 4 in PMM [29, 35, 38, 39], and 2 in women with and without headache [15, 40].

Drugs which were evaluated to manage migraine in women of reproductive age include the desogestrel progestogen-only pill (POP) [13, 14, 41, 42], extended regimen of oral CHCs [13–15, 30], oral CHCs with shortened pill-free interval [39, 43], oral CHCs with oral estrogen supplementation during the pill-free interval [44], oral CHCs with estradiol supplementation with patch during the pill-free interval [34], the combined hormonal contraceptive patch [40], the combined hormonal contraceptive vaginal ring [45], transdermal estradiol supplementation with gel [31, 32, 35], transdermal estradiol supplementation with patch [29, 33, 37, 38], transdermal estradiol supplementation with patch in women induced in pharmacological menopause [12], and the subcutaneous estrogen implant with cyclical progestogen [36].

### Desogestrel progestogen-only pill

Four studies assessed the possible benefits of the desogestrel POP in women with both MO and MA [41], MO [13, 14], or MA [42]. All studies had an observational design. The study drug was the desogestrel 75 μg/day oral pill in all studies. All the studies were performed in the setting of a reproductive clinic in women who were prescribed with the study treatments for contraception or medical reasons. Details of the studies are reported in Table 3. The quality of evidence was rated as low for all the available studies (Table 4).

**Table 3:** Studies evaluating estrogen and progestogen strategies in women of reproductive age

Study	Study design (recruitment period)	Setting (diagnostic criteria)	Women included (n)	Treatment	Duration	Outcome	Findings
Desogestrel progestogen-only pill							
Merki-Feld, 2017 [41]	Retrospective, observational, (2009–2013)	MO, MA (ICHD-2); women who required treatment for contraception or medical reasons	64; 6 dropped out (on treatment analysis)	Desogestrel 75 μg/day	90 days of observation and 90 days of treatment	Migraine days, headache intensity, days with headache score 3, analgesic use	Reduced migraine days, headache intensity, days with headache and use of pain medications
Nappi, 2011 [42]	Prospective, observational	MA (ICHD-2); women who required treatment for contraception or medical reasons	30; 2 dropped-out after 3-month (analysis on treatment at 6 months)	Desogestrel 75 μg/day	3 months of observation and 6 months of treatment	Migraine attacks, duration of aura, duration and severity of headache pain, occurrence of focal neurological symptoms or associated symptoms, analgesic use	Reduced number of migraine attacks in previous COCs users and nonusers
Desogestrel progestogen-only pill and extended regimen of combined oral contraceptives							
Morotti, 2014 [14]	Retrospective, observational (2009–2013)	MO (ICHD-2); women who required treatment for contraception or medical reasons	53; 21 dropped-out (on treatment analysis)	Desogestrel 75 μg/day vs continuous EE 20 μg plus oral desogestrel 150 μg	6 months of treatment (pre-treatment observation period not-defined)	Migraine and headache days, headache intensity, days with headache score 3, pain medication, triptan use, quality of life	Reduced migraine days, headache days pain intensity, number of days with severe pain and days with pain medication in POP users; reduced number of headache days and in days with pain medication in COCs users; reduced number of days with pain medication in the POP group compared to the COC group
Morotti 2014 [13]	Prospective, observational (2009–2013)	MO (ICHD-2) and endometriosis; women who required treatment for contraception or medical reasons	144; 27 dropped-out (on treatment analysis)	desogestrel 75 μg/day vs sequential (21/7) EE 20 μg plus desogestrel 150 μg	6 months of treatment (pre-treatment observation period not-defined)	Severity, number and duration of migraine attacks, associated symptoms	Decreased number and intensity of migraine attacks in POP users
Extended regimen of combined oral contraceptive							
Coffee, 2014 [30]	Non-randomized, open-label*	MRM without aura (modified ICHD-2 criteria); women specifically treated for headache	32; 2 dropped-out (on treatment analysis)	Extended regimen of EE 30 μg + levonorgestrel 150 μg	2 cycles of observation and 168 days of treatment	Headache severity, MIDAS score, analgesic use	Decrease in daily headache scores
Sulak, 2007 [15]	Prospective, observational	Women with and without headache (no ICHD criteria; MA excluded); women who required treatment for contraception or medical reasons	114; 12 dropped-out (on treatment analysis)	EE 30 μg plus drosperinone 3 mg	Standard 21/7-day cycles for 3 months followed by a 168-day extended placebo-free regimen	Presence and severity of headaches, analgesic use, impact of headaches on work, housework, social, recreational, and family events	Improved headache scores with the extended regimen
Combined oral contraceptives with shortened pill-free interval							
De Leo, 2011 [39]	Randomized, parallel group	PMM (ICHD-2); women who required treatment for contraception or medical reasons	60	EE 20 μg + drospirenone 3 mg 21 active + 7 placebo vs 24 active + 4 placebo	3 cycles of observation and 3 months of treatment	Duration and severity of headache	Both treatments associated with reduction in intensity and duration of attacks; greater reduction in intensity and duration in patients taking 24 active + 7 placebo vs 21 active + 7 placebo
Nappi, 2013 [43]	Non-randomized, open-label	MRM (ICHD-2); women who required treatment for contraception or medical reasons	32; 4 dropped-out (analysis on treatment on 29 women at cycle 3 and on 28 women at cycle 6)	Estradiol valerate + dienogest pill using an estrogen step-down and progestogen step-up approach 26 days + 2 placebo	3 cycles of observation and 6 cycles of treatment	Number of headache attacks, numbers of hours of headache pain, number of hours of severe headache pain, associated phenomena, analgesic use	Reduction in the number and duration of migraine attacks, in hours of severe pain, and in use of analgesics
Combined oral contraceptives with oral estradiol supplementation during the pill-free interval							
Calhoun, 2004 [44]	Retrospective and prospective, observational	MO (ICHD-1 criteria) associated with menses; indication not specified	11	EE 20 μg (days 1–21) and conjugated equine estrogen 0.9 mg (days 22–28)	1 cycle of treatment	Number of headache days, headache intensity score	Decrease in the number of headache days and in weighted headache score
Combined oral contraceptives with estradiol supplementation with patch during the pill-free interval							
MacGregor, 2002 [34]	Double-blind, placebo-controlled, randomized, crossover study	MM (ICHD-1); women specifically treated for headache	14	Estradiol 50 μg vs placebo (all patients were on combined hormonal contraceptive pill)	2 cycles of active treatment and 2 cycles of placebo	Number of pill-free intervals with migraine; number of days of migraine; severity of migraine; number of days of migraine with associated symptoms	Trend towards reducing the frequency and severity of migraine with the patch
Combined hormonal contraceptive patch							
LaGuardia, 2005 [40]	Randomized (2002–2003)	Women with and without headache; women who required treatment for contraception or medical reasons	239	EE 20 μg + norelgestromin 150 μg patch	Extended (12 weekly patch, 1 patch-free week, 3 weekly patch) vs cyclic regimen (4 cycles of 3 weekly patch and 1 patch-free week)	Headache occurrence	Less headache days in the patch on than in the patch off weeks; decrease in the headache rate during the patch-on weeks over the 16-week study period
Combined hormonal contraceptive vaginal ring							
Calhoun, 2012 [45]	Retrospective, observational (2004–2010)	Migraine with aura + MRM (modified ICHD criteria); indication not specified	28; 5 dropped out (on treatment analysis)	EE 15 μg + etonogestrel 0.120 mg	7.8 months (range: 2 to 30 months)	Aura frequency, headache frequency and intensity, resolution of MRM, headache index	Aura frequency reduced; MRM eliminated in 91.3% of subjects
Transdermal estradiol supplementation with gel							
de Lignieres, 1986 [31]	Randomized, placebo-controlled, double-blind, crossover	MM (No ICHD; migraine without aura occurring exclusively not earlier than 2 days before menstruation and no later than the last day of the menses); women specifically treated for headache	20; 2 dropped-out	Estradiol gel 1.5 mg for 7 days vs placebo	26 cycles of treatment, 27 cycles of placebo	Occurrence, duration, severity of migraine attacks, aspirin use	Reduction in the occurrence and severity of attacks and in the use of aspirin
Dennerstein, 1988 [32]	Randomized, placebo-controlled, double-blind, crossover	MM (No ICHD; regular migraine in the paramenstruum); women specifically treated for headache	22; 4 dropped-out (on treatment analysis)	Estradiol gel 1.5 mg for 7 days vs placebo	2 cycles of treatment, 2 cycles of placebo, and 1 cycle of follow-up (no treatment)	Occurrence of migraine, moderate to severe intensity migraine, analgesic use	No difference in the occurrence of attacks; reduction of moderate to severe intensity attacks
MacGregor, 2006 [35]	Randomized, double-blind, placebo-controlled, crossover	PMM or MRM (ICHD-2); women specifically treated for headache	37; 2 dropped-out (on treatment analysis)	Estradiol gel 1.5 mg for 6 days vs placebo	3 cycles of placebo, 3 cycles of treatment	Migraine days and severity, duration of attacks, associated symptoms, occurrence of aura, analgesic use	Reduction in migraine days and attacks severity; increase in migraine occurrence in the 5 days immediately after estradiol use
Transdermal estradiol supplementation with patch							
Almen-Christensson, 2011 [29]	Randomized, placebo-controlled, double-blind crossover	PMM (ICHD-2); women specifically treated for headache	38; 6 dropped-out (on treatment analysis)	Estradiol 100 μg vs placebo	2 weeks of treatment for 3 cycles for placebo and 3 cycles for active treatment	Number, severity and intensity of migraine attacks	No differences between active treatment and placebo
Guidotti, 2007 [33]	Prospective, observational	MM (ICHD-2); women specifically treated for headache	38 (10 treated with EE)	Estradiol 25 μg vs frovatriptan vs naproxen sodium	1 cycle of treatment (6 days before expected menstruation)	Number and severity of migraine attacks	Reduction in number of migraine attacks and severity of attacks with frovatriptan than with estradiol or naproxen sodium
Pradalier, 1994 [37]	Randomized, open-label study	MM (ICHD-1); women specifically treated for headache	24	Estradiol 25 μg vs estradiol 100 μg	1 cycle of observation, 2 cycles of treatment	Occurrence and severity of MM	Reduction in number of attacks with the higher dose
Smite, 1993 [38]	Randomized, placebo-controlled (1989–1990)	PMM (ICHD-1); women specifically treated for headache	20	Estradiol 50 μg vs placebo	6 days of treatment for 3 cycles (estradiol-placebo-estradiol or placebo-estradiol-placebo)	Presence, duration, severity of migraine attacks, analgesic use	No differences between active treatment and placebo
Transdermal estradiol supplementation with patch in women induced in pharmacological menopause							
Martin, 2003 [12]	Randomized, placebo- controlled, parallel group (1997–2001)	MO, MA (ICHD-1); women specifically treated for headache	23; 2 dropped-out (on treatment analysis)	goserelin 3.6 mg implant with estradiol 100 μg patches every 6 days vs goserelin 3.6 mg implant with placebo patches	1 lead-in month, 2.5 months of placebo, 1 month of goserelin injection, 2 months of randomization	Headache index, disability index, headache frequency and severity	Reduced headache and disability index and in headache frequency in the GRH agonist/estradiol group
Subcutaneous estrogen implant plus cyclical progestogen							
Magos, 1983 [36]	Retrospective, observational; women specifically treated for headache	MM with and without aura (No ICHD; attacks immediately before or during menstruation)	24	Estradiol (100 mg then decreased to 50 mg) + norethisterone 5 mg/day for 7 days per month	2.5 years (mean duration) of treatment	Improvement of menstrual migraine	95.8% of patients with improvement in MM; 46% became headache-free and 37.5% gained almost complete symptomatic relief

*MO* migraine without aura, *MA* migraine with aura, *ICHD* International Classification of Headache Disorders, *EE* ethinylestradiol

*PMM* pure menstrual migraine, *MRM* menstrually related migraine, *MM* menstrual migraine

**Table 4:**
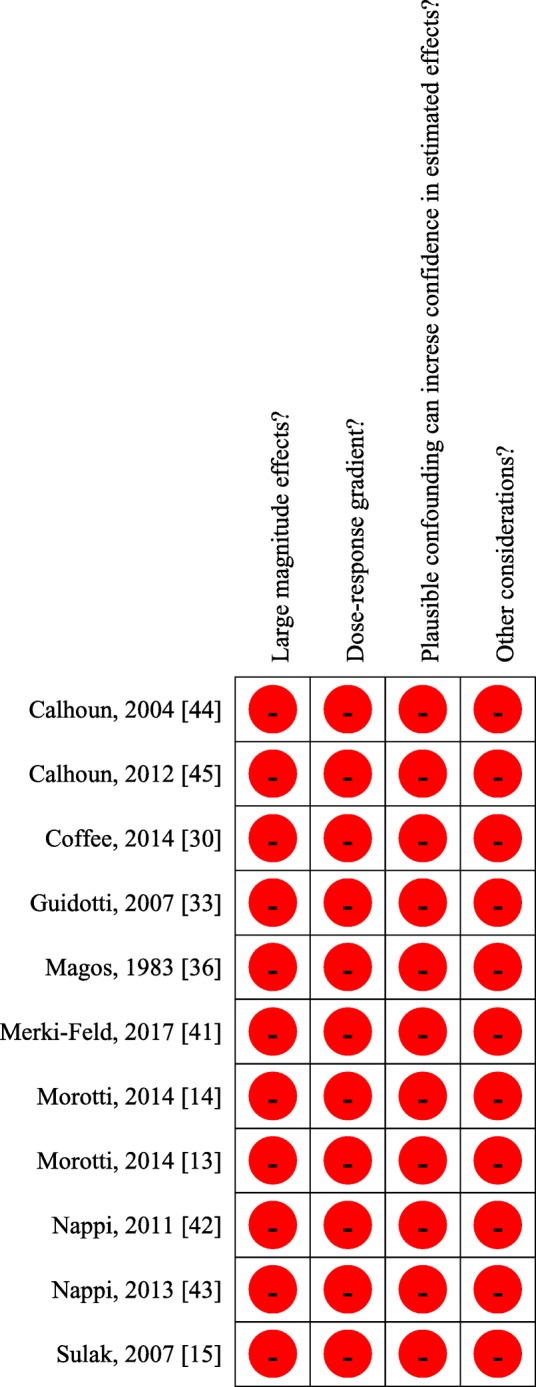
Assessment for rating up the quality of evidence for individual observational studies

Criteria for rating up the quality of evidence from observational studies were selected and addresses according to the GRADE recommendations (Guyatt et al. [26]). The red dot indicates that the study does not meet quality of evidence criterion

Merki-Feld et al. performed a retrospective, observational study in 64 women with MO and MA [41]. The diagnosis was made according to ICHD-2 criteria. There was a 90-day observation period followed by 90 days of treatment. Patients were stratified according to the use of CHCs before inclusion. Treatment with desogestrel was associated with a significant reduction in migraine days (6.2 ± 4.2 vs 5.2 ± 5.3; *P* = 0.001 in previous CHCs non users and 5.9 ± 4.1 vs 3.5 ± 3.3; *P* = 0.001 in previous CHCs users), headache intensity (17.2 ± 8.0 vs 12.7 ± 7.7; P = 0.001 in previous CHCs non users and 17.3 ± 9.3 vs 11.6 ± 8.9; P = 0.001 in previous CHCs users), days with headache score 3 (6.0 ± 5.8 vs 2.8 ± 2.7; *P* = 0.004 in previous CHCs non users and 6.7 ± 7.4 vs 2.8 ± 3.9; *P* = 0.001 in previous CHCs users), use of triptans (16.9 ± 17.8 vs 13.2 ± 12.2; *P* = 0.2 in previous CHCs non users and 9.6 ± 11.2 vs 6.1 ± 7.3; *P* = 0.003 in previous CHCs users), and MIDAS score (30.2 ± 21.8 vs 13.9 ± 15.3; P = 0.001 in previous CHCs non users and 34.7 ± 43.9 vs 20.0 ± 41.4; P = 0.001 in previous CHCs users).

Nappi et al. performed a prospective, observational study in 30 women with MA [42]. The diagnosis was made according to ICHD-2 criteria. There was a 3-month observation period followed by 6 months of treatment. The authors found a significant reduction in the number of migraine attacks (*P* < 0.001) and of aura symptoms (*P* < 0.02) with treatment. There was no benefit on the duration of headache pain and on analgesic consumption. Benefits in the reduction of migraine attacks were evident in both users (3.9 ± 1.0 vs 2.9 ± 0.8; *P* < 0.001) and non-users (3.2 ± 0.9 vs 2.6 ± 1.3; P < 0.02) of CHCs before study entry, whereas benefits on aura were evident in users of CHCs only (duration of total symptoms of aura 33.6 ± 23.3 min vs 18.6 ± 18.0 min; P < 0.02).

Morotti et al. performed a retrospective, observational study in 31 women with MO [14]. The diagnosis was made according to ICHD-2 criteria. A pre-study observation period was not defined; there was a 6-month treatment period. The authors compared pre- and post-treatment periods and additionally compared POP treatment with an extend oral CHC regimen. The authors found that treatment with desogestrel was associated with a reduction in migraine days per month (5.5 ± 2.6 vs 3.5 ± 1.2; *P* < 0.001), headache days (3.6 ± 1.5 vs 2.7 ± 1.1; *P* = 0.010), pain intensity (14.4 ± 5.4 vs 10.3 ± 2.4; *P* = 0.002), number of days with severe pain (4.9 ± 1.9 vs 3.3 ± 1.4; *P* < 0.001) and days with pain medication (6.1 ± 1.4 vs 3.5 ± 1.4; *P* < 0.001).

Morotti et al. performed an additional prospective observational study in 62 women with MO [13]. The diagnosis was made according to ICHD-2 criteria. A pre-study observation period was not defined; there was a 6-month treatment period. The authors compared pre- and post-treatment periods and additionally compared POP treatment with an extend oral CHC regimen. The authors found that treatment with desogestrel was associated with a reduction in severity on a four-point (0–3) scale (2.5 ± 0.5 vs 1.8 ± 0.7; *P* < 0.001), number (6.2 ± 2.9 vs 4.9 ± 1.7; *P* = 0.005), and duration (20.5 ± 7.9 vs 15.9 ± 4.4; P < 0.001) of migraine attacks.

Side effects associated with desogestrel use included higher headache frequency, prolonged bleeding, spotting, and acne [13, 14, 41, 42].

In conclusion, current evidence for POPs is limited as it comes solely from observational studies performed in the gynecological setting and refers only to a single agent (desogestrel pill). Available data indicate that treatment with oral desogestrel may be associated with improvement in migraine in women with MO and MA. However, treatment has been tested only in those who need it for contraception or medical reasons. The available data refer to the desogestrel pill only and no information is available for the norethisterone or levonorgestrel pills or for progestogen-only non-oral methods such as subdermal implant, depot-injection, and levonorgestrel-releasing intrauterine system. As desogestrel is safe in terms of cardiovascular risk it is a possible option even for women with MA or women with MO and additional vascular risk factors [22]. Recommendations for the desogestrel POP are reported in Table 5.

**Table 5:** Recommendations on the use of estrogens and progestogens in women of reproductive age with migraine considering their effect on migraine course

Treatment	Population	Recommendation	Quality of Evidence	Strength of Recommendation	Comments
21/7 combined contraceptive regimen with oral pill or patch*	Women with migraine who require hormonal contraception	Not suggested	None	Weak	Alternative contraceptive strategies are more convenient in migraineurs
Desogestrel-only 75 μg/day pill	Women with migraine, related or unrelated to menstruation, who require treatment for contraception or medical reasons	Suggested	Low	Weak	No evidence available for progestogen only-pills other than desogestrel 75 μg/day
	Women with estrogen withdrawal headache or worsening of the usual headache with combined hormonal contraceptives; women with new onset migraine with combined hormonal contraceptives	Suggested	None	Weak	
	Women with migraine, related to menstruation, who require migraine preventive treatments and who have contraindication or failure of conventional medical treatment	Suggested	None	Weak	
	Women with migraine, related to menstruation, who have not tried migraine preventive drugs and who have no need of desogestrel-only pill for contraception or medical reasons	Not suggested	None	Weak	
Combined oral contraceptives with shortened pill-free interval*	Women with migraine, related or unrelated to menstruation, who require treatment for contraception or medical reasons	Not suggested	Low	Weak	Data are too limited to support this option. No clear evidence that this may be better than conventional 21/7 regimen
	Women with estrogen withdrawal headache	Not suggested	None	Weak	
Combined oral contraceptives with oral estradiol supplementation during the pill-free interval*	Women with migraine, related or unrelated to menstruation, who require treatment for contraception or medical reasons	Not suggested	Low	Weak	Data are too limited to support this option. No clear evidence that this may be better than conventional 21/7 regimen. Alternative contraceptive strategies are more convenient in migraineurs
	Women with estrogen withdrawal headache	Not suggested	None	Weak	
Combined oral contraceptives with estradiol supplementation with patch during the pill-free interval*	Women with migraine, related or unrelated to menstruation, who require treatment for contraception or medical reasons	Not suggested	Low	Weak	Data are too limited to support this option. No clear evidence that this may be better than conventional 21/7 regimen Alternative contraceptive strategies are more convenient in migraineurs
	Women with estrogen withdrawal headache	Not suggested	None	Weak	
Extended regimen of combined hormonal contraceptives with pill or patches*	Women with migraine, related or unrelated to menstruation, who require treatment for contraception or medical reasons	Suggested	Low	Weak	There is no clear evidence on the preferable extended regimen (oral pill and type of pill or patch) of combined contraceptives for women with migraine Extended regimens of the contraceptive patch may be preferable over the 3 week patch + 1 patch-free week
	Women with estrogen withdrawal headache	Suggested	None	Weak	
	Women with migraine, related to menstruation, who require migraine preventive treatments and who have contraindication or failure of conventional medical treatment	Suggested	None	Weak	
	Women with migraine, related to menstruation, who have not tried migraine preventive drugs and who have no need of desogestrel-only pill for contraception or medical reasons	Not suggested	None	Weak	
Combined hormonal contraceptive vaginal ring	Women with migraine, related or unrelated to menstruation, who require treatment for contraception or medical reasons	Suggested	Low	Weak	
Transdermal estradiol supplementation with estradiol gel	Women with pure menstrual migraine	Suggested	Low	Weak	Patients should be informed that delayed migraine may occur and that treatment is potentially effective only on attacks related to menstruation
	Women with menstrually-related migraine	Suggested	Low	Weak	
	Women with estrogen-withdrawal headache	Suggested	None	Weak	
Transdermal estradiol supplementation with patch	Women with pure menstrual migraine	Not suggested	Low	Weak	
	Women with menstrually-related migraine	Not suggested	Low	Weak	
	Women with estrogen-withdrawal headache	Not suggested	Low	Weak	
Transdermal estradiol supplementation with patch in women induced in pharmacological menopause	Women with migraine	Not suggested	Low	Weak	
Subcutaneous estradiol; implant and cyclical progestogen	Women with pure menstrual migraine	Not suggested	Low	Weak	
	Women with menstrually-related migraine	Not suggested	Low	Weak	

*According to the Consensus Statement on the Safety of hormonal contraceptives in women with migraine, compounds containing estrogens are not suggested for women with migraine with aura and for women with migraine without aura and additional vascular risk factors [22]

### Extended regimen of combined oral contraceptives

Four studies assessed the possible benefits of extended regimen of oral CHC in women MRM without aura [30], in women with MO [13, 14] and in women with and without headache (excluding women with MA) [15]. Two of those studies were those by Morotti et al. which were considered for data about POP. All studies had an observational design. The study drugs were ethinylestradiol (EE) 30 μg/day + levonorgestrel 150 μg/day [30], EE 20 μg/day + desogestrel 150 μg/day [13, 14], and EE 20 μg/day + drospirenone 3 mg/day [15]. One study was performed in the setting of gynecology practice in women without an expressed need for HC and thus treatment was specifically prescribed for headache management [30]. Another three trials were conducted in the setting of a reproductive clinic in women who were prescribed with treatments for contraception or medical reasons [13–15]. Details of the studies are reported in Table 3. The quality of evidence was rated as low for all the available studies (Table 4).

Coffee et al. performed a cross-over study in 32 women with MRM without aura [30]. The diagnosis was made according to modified ICHD-2 criteria. The pre-treatment observation period was 2 menstrual cycles. During the observation period, some of the included women were on 21/7 oral CHC whereas others were not on HC. During the extended HC regimen women were randomized to frovatriptan or placebo for the treatment of acute attacks. The authors reported a decrease in average headache scores (0–10 scale) during the extended HC regimen as compared to baseline (1.29±0.10 vs 1.10±0.14; *P* = 0.034). The findings were consistent in women taking or not taking HCs during the observation period. Users of extended HC regimen also had a decrease in MIDAS scores as compared to baseline.

Morotti et al. performed a retrospective, observational study in 22 women with MO [14]. The authors found that the extended regimen of CHC was associated with a reduction in the number of headache days (3.1 ± 0.9 vs 2.4 ± 1.9; *P* = 0.029) and in days with pain medication (6.1 ± 1.4 vs 4.2 ± 1.3; *P* = 0.037) but not with a reduction in pain intensity or use of acute medications.

Morotti et al. performed a prospective observational study in 82 women [13]. The authors compared pre- and post-treatment periods. The authors found that the extended regimen of combined oral contraceptive was associated with a reduction in the duration of migraine attacks (22.7 ± 9.0 vs 18.9 ± 6.0; *P* = 0.007) but not in reduction of pain severity and number of attacks.

Sulak et al. performed a prospective observational study in 114 women with and without headache [15]. Women with MA were excluded. The diagnosis was not made according to ICHD-2 criteria. The authors compared a standard 21/7 day pill cycle for 3 months followed by a 168-day extended placebo-free regimen. The authors found that during the first 28 days of the extended placebo-free regimen, daily headache scores (measured with the Penn Daily Symptom Rating) decreased from 0.5 (standard error [SE] 0.05) to 0.3 (SE 0.04; *P* < 0.0001) and average number of daily pain pills decreased from 0.6 (SE 0.08) to 0.3 (SE 0.05; *P* = 0.003) compared with the standard 21/7 regimen. The difference persisted throughout the remainder of the 168-day regimen.

Reported side effects of the extended oral CHC included irregular bleeding, breast tenderness and mood swings [13–15].

In conclusion, current evidence on the use of extended oral CHCs regimens in women with migraine is limited as it comes from observational studies performed in the gynecological setting [13–15]. In only one study only treatment was specifically used for headache [30]. Available data refer to EE 30 μg + levonorgestrel 150 μg [30], oral EE 20 μg + oral desogestrel 150 μg for 6 months [13, 14], and to EE 30 μg + drospirenone 3 mg for 160 days [15]. One study only provided comparison of the extended regimen with conventional 21/7 regimen. This study included women with and without headache and supported greater benefits of the extended regimen over the conventional one. No studies evaluated whether the use of extended regimens in women who are in the conventional 21/7 regimen and who experience attacks during the pill-free period may reduce the burden of those attacks. As available data point toward possible benefits of extended regimen of CHCs in women with MO, this possibility should be considered in women who require the use of CHCs. Recommendations for the extended regimen of combined oral contraceptives are reported in Table 5.

### Desogestrel progestogen-only pill vs extended regimen of combined oral contraceptives

Two of the studies which addressed, in women with MO, the POP and extended regimen of oral CHC, also compared the two treatments [13, 14]. The study drugs were desogestrel 75 μg/day oral pill versus EE 20 μg/day + desogestrel 150 μg/day. Details of the studies are reported in Table 3. The quality of evidence was rated as low for all the available studies (Table 4).

Morotti et al. compared data of 31 women with MO who were treated with desogestrel 75 μg/day and 22 women with MO who were treated with continuous ethinylestradiol (EE) 20 μg/day + desogestrel 150 μg/day [14]. The authors found that desogestrel 75 μg/day as compared to the continuous EE 20 μg/day + desogestrel 150 μg/day was associated with a reduction in the number of days with pain medication (3.5 ± 1.4 vs 4.5 ± 1.5; *P* = 0.044). There was no difference between the two group in migraine days, headache days, headache intensity, days with headache score 3 and triptan use.

Morotti et al. compared data of 62 women with MO who were treated with desogestrel 75 μg/day and 82 women with MO who were treated with continuous EE 20 μg/day + desogestrel 150 μg/day for 6 months [13]. The authors found that desogestrel 75 μg/day as compared to the continuous EE 20 μg/day + desogestrel 150 μg was associated with a reduction in pain severity (1.8 ± 0.7 vs 2.2 ± 0.5; *P* < 0.001), duration of attacks (15.9 ± 4.4 vs 18.9 ± 6.0; *P* < 0.001) and number of attacks (4.9 ± 1.7 vs 5.9 ± 2.1; *P* < 0.001).

In conclusion, current evidence referring to the benefits of POP as compared to extended regimen of oral CHD are limited. The available data come from two observational studies performed in the gynecological setting and refer only to desogestrel versus EE + desogestrel. Available data suggest more benefit from desogestrel over the oral CHC but evidence is too preliminary to draw firm conclusions.

### Combined oral contraceptives with shortened pill-free interval

Two studies assessed the role of CHC with shortened pill-free interval in women with MO associated with menstruation [43] or in women with PMM [39]. One study had an interventional design [39]; one was observational [43]. Study regimens were EE 20 μg + drospirenone 3 mg for 24 days + 4 placebo days versus EE 20 μg + drospirenone 3 mg for 21 days + 7 placebo days [39], and estradiol valerate + dienogest using an estrogen step-down and progestogen step-up approach for 26 days + 2 placebo days [43]. The two studies were performed in the setting of a reproductive clinic in women who were prescribed treatment for contraception or medical reasons [39, 43]. Details of the studies are reported in Table 3. The quality of evidence was rated as low for all the available studies (Table 4 and Table 6).

**Table 6:**
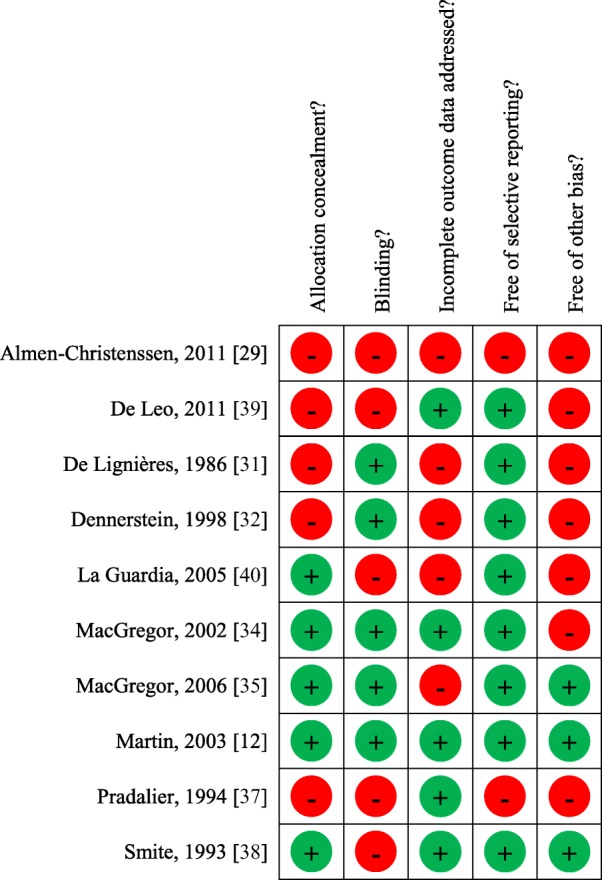
Rating of the quality of evidence for individual interventional trials

Quality of evidence for the individual randomized trials was rated according to the GRADE recommendations (Guyatt et al. [25]. The green dot indicates that the study meets quality of evidence criterion and the red dot that the study does not meet quality of evidence criterion. Where studies did not report information about the individual criterion, this was considered as not meet

De Leo et al. performed a randomized, parallel group study in 60 women with PMM without aura [39]. The diagnosis was made according to ICHD-2 criteria. Treatment duration was 3 months and before enrollment headaches were tracked for 3 menstrual cycles. Both treatment with the conventional and the shortened pill-free interval were associated with reduction in headache intensity and days of migraine as compared to baseline observation. Treatment with the shortened pill-free interval was associated with significantly greater reduction in the intensity and duration of MM as compared to 21/7 HC. Improvements were observed from the first cycle and increased over the study period. Of note this study did not provide numbers to quantify benefits of treatment but results were available as figures only. No side effects were reported in the study.

Nappi et al. performed an open-label observational study in 32 women with MRM [43]. The diagnosis was made according to ICHD-2 criteria. Pre-treatment observation period was 3 menstrual cycles; duration of treatment was 6 menstrual cycles. The study included a group of women who had never used CHCs and a group who had used them and stopped them because of exacerbation of MRM at least 3 months before enrollment. After treatment, the number of migraine attack days was reduced from 2.7 ± 0.9 days at baseline to 2.2 ± 0.7 (*P* < 0.001) at cycle 3 and 2.0 ± 0.7 (*P* < 0.001) at cycle 6. Similarly, the duration of headaches was reduced from 44.7 ± 13.5 h at baseline to 24.7 ± 10.1 h (*P* < 0.001) at cycle 3 and remained reduced 24.1 ± 9.2 h (*P* < 0.001) at cycle 6. The numbers of hours of severe pain was reduced from the baseline of 21.9 ± 7.4 h to 15.4 ± 4.9 h (P < 0.001) at cycle 3 and continued at the lower levels of 15.0 ± 5.0 h (*P* < 0.001) at cycle 6. A significant reduction in the number of analgesics use was observed from baseline of 4.7 ± 1.1 to 3.3 ± 0.7 (*P* < 0.001) at cycle 3, which dropped even lower to 2.9 ± 0.6 by cycle 6 (*P* < 0.001 cycle 3 versus cycle 6). Benefits were observed in both users and non-users of combined oral contraceptives before study entry. The only side effect reported in this study was spotting. Some women in this study had to stop the HC because of worsening of the migraine.

In conclusion, current evidence on the use of oral CHCs with shortened pill-free interval in women with migraine is limited. Available studies included PMM or MRM. One study suggested superiority of the shortened pill-free interval treatment over the conventional one [39]. Available studies are heterogeneous referring to adopted regimen and there is no evidence which may indicate superiority of one regimen over the other possibilities. Additionally, evidence mostly support the use of CHCs with shortened pill-free interval in women who need them for contraceptive or gynecological reasons. But there is not enough evidence to use this treatment solely for the management of migraine. The extended regimen is safe and not associated with significant adverse events, but it is important to note that some women with migraine may be particularly sensitive to hormonal administration and need to stop treatment because worsening of headache [43]. It may be difficult to establish if the worsening is really related to treatment or rather to natural fluctuations in the migraine course nor it is clear if improvements can be observed with continuation of treatment. Recommendations for the combined oral contraceptives with shortened pill-free interval are reported in Table 5.

### Combined oral contraceptives with oral estrogen supplementation during the pill-free interval

One observational study assessed the role of CHC with oral estrogen supplementation during the pill-free interval in women with MO associated with menstruation and women with migraine associated with CHC withdrawal bleeding [44]. The study regimen was EE 20 μg (days 1–21) + conjugated equine estrogens 0.9 mg (days 22–28). In this study patients were recruited both from a headache center and from gynecology practice.

Calhoun performed a cross-over prospective and retrospective study in 11 women with MO associated with menstruation or withdrawal bleeds. The diagnosis was made according to modified ICHD-2 criteria. Duration of active treatment was for one cycle only. During the pre-treatment observational phase some of the included patients were not using hormone therapy (*n* = 3), whereas most of them (*n* = 8) were on hormonal treatment. There was heterogeneity regarding doses, duration, and type of treatments. The duration of the observational period before intervention was not reported. The authors found that all patients achieved a 50% reduction in the number of headache days. Mean number of headache days per month was 7.6 at baseline and 1.6 after treatment (76.3% reduction). Also headache intensity score substantially improved (77.9% decrease). No information on side effects and adverse events was reported.

In conclusion, evidence on the use of oral CHCs with oral estrogen supplementation during the pill-free interval in women with migraine is limited to a single unreliable study. Recommendations for the combined oral contraceptives with oral estrogen supplementation during the pill-free interval are reported in Table 5.

### Combined oral contraceptives with estradiol supplementation with patch during the pill-free interval

One interventional study assessed the possible benefits of transdermal estradiol supplementation with patch in women with migraine during the pill-free interval of combined oral contraceptives [34]. The study drug was 50 μg estradiol patch specifically prescribed for headache management. Details of the study is reported in Table 3. The quality of evidence was rated as low (Table 4 and Table 6).

MacGregor et al. performed a double-blind, placebo-controlled, randomized, cross-over study in 14 women [34]. The authors addressed as active treatment estradiol 50 μg/24 h transdermal patch. The active treatment was compared with placebo. All women included in the study were taking a CHC pill. The estradiol patch was used for 2 cycles during the pill-free week and the placebo patch was used for additional 2 cycles during the pill-free week. Authors were unable to meet their recruitment target of 20 women. There was no significant reduction in number of pill-free intervals with migraine, number of days of migraine, severity of migraine, number of days with migraine with associated symptoms.

Adverse events related to transdermal estradiol supplementation were changes in withdrawal bleeding patterns reported by three women.

As these data from a single study show no benefit of estradiol supplementation with patches on migraine occurrence during the pill-free interval, this cannot be considered as a possible therapeutic option. Recommendations for the transdermal estrogen patch supplementation during the pill-free interval are reported in Table 5.

### Combined hormonal contraceptive patch

One study assessed the possible benefits of the CHC patch in women with and without headache [40]. The study had an interventional design. The study drug was EE 20 μg + norelgestromin 150 μg/24 h patch. The study was performed in the setting of reproductive clinics in women who were prescribed with the study treatments and not specifically for headache management. Details of the study are reported in Table 3. The quality of evidence was rated as low for this study (Table 6).

LaGuardia et al. performed a prospective observational study in 239 women with and without headache; women with MA were excluded [40]. The authors compared a cyclic regimen (4 cycles of 3 weekly patch applications and 1 patch-free week) with an extended regimen (12 weekly patch applications, 1 patch-free week, 3 weekly patch applications). The authors found that, across both regimens, the mean number of headache days per week were 0.63 when the patch was on and 1.19 when the patch was off (*P* < 0.001). Moreover, the headache rate during the patch-on weeks in both regimens decreased significantly over the 16-week study period (*P* = 0.0002); this reduction was consistent for the two regimens. The study did not report the side effects associated with the use of the patch.

In conclusion, data referring to the effect of CHC patch on migraine are very limited. One study only evaluated the effect of the CHC patch on headache course [40]. Data from this study suggested that there is a relationship between hormone withdrawal and headache occurrence. The mean headache days was higher during the patch-off week. In the patch-off week following the extended regimen the increase in headache frequency did not exceed that seen at baseline. Recommendations for the combined hormonal contraceptive patch are reported in Table 5.

### Combined hormonal contraceptive vaginal ring

One study assessed the possible benefits of the vaginal ring in women with MA associated with menstruation [45]. The study had an observational design. The study drug was EE 15 μg + etonogestrel 0,120 mg vaginal ring. The study was performed in the setting of subspecialty clinic devoted to hormonal issues in women’s headaches. It is not clearly indicated if treatment was prescribed for contraception or medical reasons or specifically for headache management. Details of the study are reported in Table 3. The quality of evidence was rated as low for this study (Table 4).

Calhoun et al. performed a retrospective, observational study in 28 women with MA associated with menstruation [45]. The diagnosis was made according to modified ICHD-2 criteria. Eight women used the ring continuously without interruption, 15 used the ring for 12 consecutive weeks, followed by 1 week of 0.075 mg transdermal 17β estradiol patches, 5 stopped the ring. Ten of the women were already taking HCs before enrollment. Headache frequency was monitored with diaries. Pre- and post-treatment observation periods were not defined and authors did not provide any information about migraine frequency, severity or disability before and after treatments. The authors reported that, after a mean observation of 7.8 months, the use of vaginal ring eliminated MRM in 91.3% of subjects. They further reported single patient data showing the course of aura over the study period and those data suggest improvements in aura in most of the included patients. Five of the included patients discontinued treatment for nausea (*n* = 2), for ring expulsion (n = 2), and for facial swelling and abdominal pain (*n* = 1).

In conclusion, current evidence on the use of CHC vaginal ring in women with migraine is very limited. There are not enough data to clarify if the use of CHCs by vaginal ring may improve migraine. The only available study did not provide sufficiently rigorous information to fully understand benefits and disadvantages of treatment. Additionally, the study included women with MA, a condition in which the use contraceptives containing estrogens are contraindicated because concerns over a possible increase in the risk of ischemic stroke [22]. No ischemic strokes were reported during the study period but the number of included patients was low and the duration of follow-up was too short to draw conclusions. Recommendations for the combined hormonal contraceptive vaginal ring are reported in Table 5.

### Transdermal estradiol supplementation with gel

Three studies assessed the role of transdermal estradiol supplementation with gel in women with MM [31, 32] and in women with PMM or MRM [35]. All studies had an interventional design. Two studies were randomized [32, 35]. The study drug was estradiol gel 1.5 mg for 6 [35] or 7 days [31, 32]. In all the studies, transdermal estradiol gel was evaluated against placebo [31, 32, 35]. In all the studies treatment was specifically prescribed for headache management. Details of the study are reported in Table 3. The quality of evidence was rated as low for the two studies [31, 32], high for one of the studies [35] and low for the overall evidence (Table 6).

De Lignieres et al. performed a randomized, placebo-controlled, double-blind, crossover study in 20 women with MM [31]. The diagnosis was not made according to ICHD criteria. The authors reported a reduction in the occurrence and severity of attacks and in the use of aspirin. Menstrual attacks occurred in 30.8% of the estradiol cycles and in 96.3% of the placebo cycles (*P* < 0.01). Attacks that occurred during estradiol treatment were considerably milder and shorter than those occurring during placebo treatment and were associated with less use of aspirin. One out of 20 patients had migraine three days after stopping estradiol treatment.

Dennerstein et al. performed a randomized, placebo-controlled, double-blind, cross-over study in 22 women with MM [32]. The diagnosis was not made according to ICHD criteria. The authors reported no difference in the occurrence of all attacks, but a reduction of moderate to severe intensity attacks. Overall there were no significant differences in the occurrence of migraines between pre- and post-treatment cycles. There was a significant difference in the occurrence of moderate to severe intensity migraine during the months of treatment with percutaneous estradiol compared with placebo gel (*t* = 2.67; *P* < 0.05). As the authors considered that treatment would not be expected to affect migraine which occurred prior to or after the use of the gel, they performed a further analysis with these migraines omitted. They found that the alleviation of headaches by percutaneous estradiol compared with placebo was then significant (*t* = 3.96; *P* < 0.001). Additionally, significantly less medication was utilized during percutaneous estradiol use, compared with placebo.

MacGregor et al. performed a randomized, double-blind, placebo-controlled, crossover study in 37 women with PMM or MRM [35]. The diagnosis was made according to ICHD-2 criteria. The authors reported a reduction in migraine days and attack severity. Estradiol was associated with a 22% reduction in migraine days per woman (relative risk [RR] 0.78; 95% CI 0.62 to 0.99). These attacks were also less severe (*P* = 0.03) and there was evidence that they were associated with less nausea, although this difference was not significant. However, in this study authors found an increase in migraine occurrence in the 5 days immediately following estradiol use compared to placebo (RR 1.40; 95% CI 1.03 to 1.92, P = 0.03). Of the 22 women who benefited from using the estradiol gel (they had fewer migraines compared to using placebo), 15 experienced post-gel migraine (they had more migraine days during the 5 days after the estradiol gel compared to the 5 days after placebo). In this study a fertility monitor was used to predict menstruation and hence to indicate when to apply the gel.

In the available studies, no serious adverse events were reported associated with the use of estradiol gel. Some of the patients experienced cutaneous rash, anxiety, or amenorrhea. Estradiol use was also associated with an increase in the length of the follicular phase [35].

In conclusion, current evidence on the use of transdermal estradiol supplementation with gel in women with migraine is limited. Transdermal estradiol supplementation with gel is easy and well tolerated, thus offering considerable advantages over other strategies of estrogen administration. One challenge is to predict when to apply the gel especially in women with irregular menstruation. Fertility monitors may be useful but are not always practical in daily life. Possible benefits may be offset by an increase in migraine following gel use, associated with an iatrogenic delayed estrogen withdrawal. Possible reasons for the occurrence of post-gel estrogen withdrawal migraine are that the dose of estradiol was inadequate; the duration of treatment was too short; or perhaps that exogenous estrogen inhibits the follicular rise of endogenous estrogen. Extending the duration of use of estradiol supplements until endogenous estrogen had risen might prevent post-supplement estrogen withdrawal migraine. However, there is no evidence to support this strategy so far due to the paucity of data and the possibility of delayed migraine, estrogen supplementation cannot represent a first-line therapy in women with MM. However, this option may be considered when other strategies have failed or are not feasible. Women should be aware that delayed migraine may occur and in those circumstances treatment should be withheld. Recommendations for the transdermal estradiol supplementation with gel are reported in Table 5.

### Transdermal estradiol supplementation with patch

Four studies assessed the possible benefits of transdermal estradiol supplementation with patch in women with PMM [29, 38] or MM [33, 37]. Three studies were interventional [29, 37, 38] and one was observational [33]. The study drug was estradiol patch releasing from 25 to 100 μg/24 h. In all the studies treatment was specifically prescribed for headache management. Details of the studies are reported in Table 3. The quality of evidence was rated as low for all the available studies (Table 4 and Table 6).

Almen-Christensson et al. performed a randomized, placebo-controlled, double-blind, crossover study in 38 women with PMM [29]. The diagnosis was made according to ICHD-2 criteria. The authors addressed as active treatment estradiol 100 μg/24 h transdermal patch. The treatment was compared with placebo. Women started treatment 7 days before the estimated onset of menstrual bleeding and continued for two weeks; this was repeated for three consecutive menstrual cycles. The study was prematurely stopped because of difficulties in recruitment. Authors did not find any benefit in number, severity, and intensity of migraine attacks.

Guidotti et al. performed an observational, prospective, parallel group, open-label study in 38 women with MM [33]. The diagnosis was made according to ICHD-2 criteria. The authors addressed as active treatments estradiol 25 μg transdermal patch or frovatriptan or naproxen sodium. Each treatment was started 2 days before expected onset of menstrual headache and continued for 6 days for a single cycle. Authors reported that frovatriptan was associated with reduced migraine incidence and severity while they did not report benefits from transdermal estradiol.

Pradalier et al. performed a randomized, open-label study in 24 women with MM [37]. The diagnosis was made according to ICHD-1 criteria. The authors studied two different doses of the estradiol patch (25 vs 100 μg/24 h). There was 1 cycle of observation and 2 cycles of treatment. The authors found that the high dose was associated with no attacks or decreased number of attacks in 8/12 patients while the low dose was associated with no attacks or decreased number of attacks in 2/12 patients. MM occurred in 11/12 patients treated with the low dose and in 6/12 patients treated with the high dose. More patients used rescue treatments with the low dose (10 patients) than with the high dose (4 patients; *P* < 0.05).

Smite et al. performed a randomized, placebo-controlled study in 20 women with PMM [38]. The diagnosis was made according to ICHD-1 criteria. The authors addressed as active treatment estradiol 25 μg/24 h transdermal patch. The treatment was compared with placebo. There were 6 days of treatment for each cycle (estradiol-placebo-estradiol or placebo-estradiol-placebo). The authors did not find any difference in presence, duration, and severity of migraine attacks and in the use of analgesics between active treatment and placebo.

Adverse events related to transdermal estradiol supplementation occurred in a variable proportion across studies and were not serious. They consisted of increased headache, local skin reactions, nausea, early bleeding and increased blood pressure [29, 33, 37, 38].

In conclusion, current evidence on the use of transdermal estradiol supplementation with patch in women with MM or PMM is limited. As the aim of transdermal estradiol supplementation is to maintain estradiol concentrations stable at the time of the anticipated start of the bleeding and migraine attacks, dosing and timing of treatment are very important. Authors of the available studies pointed out that 25 and 50 μg of estradiol supplementation may be too low to achieve clinical benefits whereas higher doses may increase risk of ischemic stroke [33]. Also, duration of estradiol supplementation may impact on efficacy. Limitations of transdermal estradiol supplementation may also include difficulties in establishing the time of ovulation. As there are currently no data showing benefits of estradiol supplementation with patches on migraine occurrence, this cannot be considered as a possible therapeutic option. Recommendations for the transdermal estradiol supplementation with patch are reported in Table 5.

### Transdermal estrogen supplementation with patch in women with induced in pharmacological menopause

One study assessed the possible benefits of transdermal estrogen supplementation with patch in women with MO and MA, in whom a pharmacological menopause was induced [12]. The study had an interventional design. Treatment was specifically prescribed for headache management. Details of the study are reported in Table 3. The quality of evidence was rated as high for this study (Table 6). The overall quality of evidence for the specific treatment was considered as low, because of evidence was not replicated by other studies.

Martin et al. performed a randomized, placebo controlled, parallel group study in 23 women with MO and MA [12]. The diagnosis was made according to ICHD-1 criteria. All patients received a 3.6 mg goserelin implant to induce medical oophorectomy. Thereafter women were randomized to estradiol 100 μg/24 h every 6 days vs placebo patches. The study was performed in the setting of headache and internal medicine clinic. There was a 2-month randomization period. The authors found a 34% improvement (*P* = 0.025) in the headache index, a 24% improvement (*P* = 0.003) in headache severity, and a 39% improvement (*P* = 0.035) in disability during treatment as compared to pre-treatment phase. No improvements were observed in women allocated to placebo. Adverse events were reported in 9 women and included urticaria, worsening depression, and worsening headaches.

In conclusion, evidence referring to the use of transdermal estradiol supplementation in women induced in pharmacological menopause is very limited, but a single study showed some benefits [12]. This condition is associated with risk of osteoporosis, depression, irritability, hot flashes, decreased libido and vaginal dryness which however could be minimized by estrogen add-back therapy. Medical menopause associated with estradiol treatment is not appropriate for most women of reproductive age but future studies need to address its utility in women who have worsening of their headache during the perimenopause. Recommendations for the transdermal estradiol supplementation with patch in women induced in pharmacological menopause are reported in Table 5.

### Subcutaneous estrogen implant plus cyclical progestogen

One study [36] assessed a subcutaneous estrogen implants in women with MM. The study had an observational design. Treatment was specifically prescribed for headache management. Details of the study are reported in Table 3. The quality of evidence was rated as low (Table 4).

Magos et al. performed a retrospective, observational, cross-over study in 24 women with MM with and without aura [36]. The diagnosis was not made according to ICHD criteria. Authors addressed as active treatment an estradiol implant (100 mg then decreased to 50 mg) plus norethisterone 5 mg/day for 7 days per month to induce menstruation. The implants were inserted in the subcutaneous fat of the lower abdominal wall. Patients were included in this study specifically to treat migraine after failure of previous treatments. Mean duration of treatment was 2.5 years. Pre- and post-treatment observation periods were not defined and authors did not provide any information about migraine frequency, severity or disability before and after treatment. No use of headache diaries was reported. In this study, all but one patient noted an improvement in their menstrual migraine following treatment. Eleven (46%) became completely headache-free and nine (37.5%) gained almost complete symptomatic relief. All these patients were able to reduce or stop previous therapy and considered the implant treatment to be the most effective. Three patients (12–5%) reported partial relief, and one patient (4%) gained no benefit. No adverse events or side effects were reported.

In conclusion, current evidence on the use of subcutaneous estrogen implants in women with MM is very limited. In women with MM, there are not enough data to assess if the use of the subcutaneous implants may improve migraine. The only available study did not provide information in a sufficient rigorous way to fully assess benefits and disadvantages of treatment. Due to the promising benefits reported by the single available pilot study, further data are needed to evaluate possible advantages related to treatment. Recommendations for a subcutaneous estrogen implant plus cyclical progestogen are reported in Table 5.

## Discussion

Our systematic review revealed that available evidence referring to the use of estrogens and progestogens in women of reproductive age and their effect on migraine is limited. All the recommendations were based on low quality evidence. There is no evidence on how to manage women with headache attributed to the use of estrogens. The strength of recommendation was rated as low in all cases. Further, most of the contraceptive hormone strategies were evaluated in the gynecological setting in women who required estrogens and/or progestogens for contraception or medical reasons. Much more limited is the evidence on the use of those drugs in the headache setting in women who do not require them for gynecological reasons.

Available data, albeit very weak, indicate that the desogestrel 75 μg /day pill is the option which may offer more evident benefit on migraine course. From a cardiovascular point of view in women with migraine it is also the safest form [22, 46]. The desogestrel pill may improve migraine frequency and severity in women with MA or MO not necessarily related to menstruation. However, it has been addressed only in women who required it for contraception or medical reasons and no studies specifically aimed to evaluate this drug as a specific therapeutic option for women with migraine is available. It is important to note that in some patient the desogestrel pill may worsen migraine or may be associated with new onset migraine. Unfavorable bleeding patterns are a common cause for cessation of treatment [6].

The extended regimen of CHC either oral or with the patch is a further strategy to consider in women with migraine. As compared to regimens with hormone-free intervals, this strategy appears more beneficial. However, even in this case data are preliminary and evidence is limited to women who require CHC for contraception or medical reasons. This option is not suggested for women with MA or for women with MO and additional vascular risk factors. Although no studies addressed specifically the issue, Panelists suggested trying this option for women with estrogen withdrawal headache, particularly those women already experiencing headache or migraine during the pill-free interval. Less certain is the option of a shortened pill-free interval. No eligible studies were found evaluating the effects of the conventional 21/7 oral CHC. This is probably because observational epidemiological studies indicated that this contraceptive method is associated with de novo occurrence of migraine or migraine worsening in a substantial proportion of female migraineurs, although no studies record whether the attacks occur during pill taking or during the pill-free interval [47–50]. It is important to note that migraine may improve in some patients with the use of oral CHC [51], but unfortunately there are no tools or clinical features to predict the course of migraine after oral CHC initiation.

In women who have menstrual attacks of migraine during natural cycles, a therapeutic option may the administration of estradiol gel. However, this option may be associated with delayed headache and is feasible only in those who have predictable bleeding and predictable migraine. Estradiol supplementation with patches is a further possibility. However, as evidence referring to the optimal dose of the patch is unclear, it is not suggested.

In conclusion, this statement provides evidence-based and expert-agreed guidance to clinicians for the management of migraine with exogenous estrogens and progestogens. The available evidence is weak and further research in needed to clarify which are the best estrogens and progestogens options in women with migraine. Further studies should also establish whether the treatments discussed may be specifically applied to treat migraine in selected women.

## Abbreviations

CHC: Combined hormonal contraceptives
CI: Confidence interval
EE: Ethinylestradiol
EHF: European Headache Federation
ESCRH: European Society of Contraception and Reproductive Health
HC: Hormonal contraception
ICHD: International Classification of Headache Disorders
MA: Migraine with aura
MM: Menstrual migraine
MO: Migraine without aura
MRM: Menstrually related migraine
PHC: Progestogen-only hormonal contraceptive
PMM: Pure menstrual migraine
POP: Progestogen-only pill
RR: Relative risk
SE: Standard error
